# Reliability Measurement for Mixed Mode Failures of 33/11 Kilovolt Electric Power Distribution Stations

**DOI:** 10.1371/journal.pone.0069716

**Published:** 2013-08-01

**Authors:** Faris M. Alwan, Adam Baharum, Geehan S. Hassan

**Affiliations:** 1 School of Mathematical Sciences, University Sains Malaysia, Penang, Malaysia; 2 School of Computer Sciences, University Sains Malaysia, Penang, Malaysia; 3 Statistics Department, College of Administration and Economics, Baghdad University, Baghdad, Iraq; 4 Ibn-Rushud College of Education, Baghdad University, Baghdad, Iraq; University of Adelaide, Australia

## Abstract

The reliability of the electrical distribution system is a contemporary research field due to diverse applications of electricity in everyday life and diverse industries. However a few research papers exist in literature. This paper proposes a methodology for assessing the reliability of 33/11 Kilovolt high-power stations based on average time between failures. The objective of this paper is to find the optimal fit for the failure data via time between failures. We determine the parameter estimation for all components of the station. We also estimate the reliability value of each component and the reliability value of the system as a whole. The best fitting distribution for the time between failures is a three parameter Dagum distribution with a scale parameter 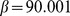 and shape parameters 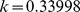 and 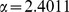. Our analysis reveals that the reliability value decreased by 38.2% in each 30 days. We believe that the current paper is the first to address this issue and its analysis. Thus, the results obtained in this research reflect its originality. We also suggest the practicality of using these results for power systems for both the maintenance of power systems models and preventive maintenance models.

## Reliability and Failure Functions

 Electric power is transmitted through an electric circuit. The terms “high voltage” and “high power” indicate that the voltages of the electrical energy are high enough to inflict harm or death on living things. Electrical power systems are highly complex and extremely integrated. Reliability is one of the most important factors considered in the planning, design, operation, and maintenance of electric power systems [1], [2]. This factor is one of the most effective indicators of product quality that buyers take into account when choosing among different varieties [3]. Moreover, reliability generally becomes more important to consumers, as failure, repair, and maintenance entail expensive costs [3]. The reliability function is a mathematical and engineering indicator that is used to describe the state of the equipment in the system through the probability function. Many factors and definitions are related to reliability (e.g., mean time to failure [MTTF], mean time between failures [MTBF], and mean time to repair [MTTR]). The MTTF is the expected value representing the return period of equipment failure [4]–[7]. It can be expressed mathematically as [8], 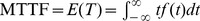, where *E(t)* is the expected value of time, and *f(t)* is the probability density function (pdf) for variable t. The MTTF is also referred to as the expected life. The mean time between failures (MTBF) and the MTTR are defined in Section Availability. The term *reliability* can be defined in many ways. For example, for an electrical switch, reliability may be defined as the probability that it successfully functions under a stipulated load and at a specific temperature. An operational definition of reliability must be sufficiently precise to establish a clear distinction between reliable and unreliable items. In addition, this definition must be sufficiently general to account for the complexities that arise in making this determination [8]. Based on this definition of reliability, reliability analyses often involve the analysis of binary outcomes *(0, 1)* (i.e., success = 1, failure data = 0) [8].

Assume that the period of failure *T* is a continuous random variable with values in a positive real line. Many methods are available to specify the properties of a random variable [8]. The first method involves using the pdf, *f(t)*, that satisfies, 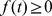 and 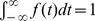.

When *T* is a Dagum random variable, its *pdf* is [9]–[11]

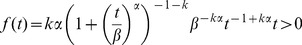
(1)in which 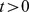, 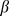 is the scale parameter (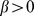 ), 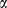 (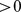) and 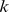 (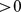) are the shape parameters.

 A second method to specify the properties of T is the *cumulative distribution function*. Mathematically, this function is expressed as [12].
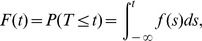



where *f(s)* is a pdf. The *cumulative distribution function* is the complement of the *reliability function*, and thus, it is called the *unreliability function* [8]. The *cumulative distribution function* for a Dagum random variable is



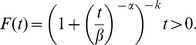
(2)A third method to specify the properties of a random variable is through its *reliability function*, also known as the *survival function* [8]. We define the *reliability function* as



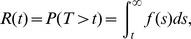
where *f(s)* is a pdf. The *reliability function* for a Dagum random variable is [9], [13], [14].
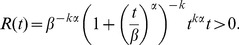
(3)


The fourth method specifies the properties of a random variable as the *hazard function*, also called the *instantaneous failure rate function* (further details are provided in [8]).
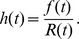



The *hazard function* for a Dagum random variable is [14]

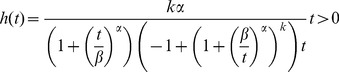
(4)


The functions *f(t)*, *F(t)*, *R(t)*, and *h(t)* are called “failure functions.”

## Availability

At first, we have to define several factors that are closely associated with availability (e.g., failure, availability, and so on). Failure is defined as the incapability of the system (subsystem or one of its components) to perform its job [15], [16] or the “inability of the item to meet the requirements of the work”[17], [18]. The term “available” is defined as the state of an item such that it can perform its function under stated conditions of use and maintenance in the required location [19]. Most researchers define availability as the probability that an item will be available [19], [20] or the probability that the system will operate satisfactorily at any point in time when operating under a specified condition [20], [21].
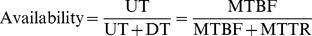



where UT is the uptime or operating time, DT is downtime (excluding free time), MTBF is the mean time between failures, and MTTR is the mean time to repair. For a more accurate quantity, “inherent availability” is defined as [19], [21]:
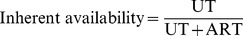
where ART is the active repair time. The MTBF is defined by Frankel, Dinesh and Bryant [15], [22], [23] as a parameter of basic reliability of the repairable components. It is the ratio of the total number of life units for components of the total number of failures. MTTR is the mean time to repair, it is defined as the whole time required to manage the failure, including factors: the way in which the fault is detected and the response speed of the maintenance team with the repair time [24]. The mean corrective maintenance time is defined by Dhillon [25] as the main criterion of the maintainability of repairable items. It represents the average (mean) time required to repair failed equipment. This criterion can be observed based on the inherent availability equation wherein availability is integrated between reliability and the times for maintenance and repair [26]. This relationship is very important because it is used to express the probability that the system will be operating according to the mission time without failure [26].

## Description of the Problem

The electric power distribution station in Iraq was designed to have two power transformers (T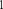 and T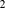). Each transformer has a circuit breaker with limited capacity (1,200 A), denoted as CBT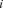 functions as a main circuit breaker of the transformer. These two transformers are connected to the communication bus situated between them (termed as Bus-Bar). The Bus-Bar feeds group of feeders (10 feeders). The Bus-Bar was divided into two parts separated by circuit breaker with a limited capacity of 800 A, called the Bus-Bar circuit breaker (CBB). Each one of the ten feeders has a circuit breaker with a limited capacity of 400 A, called as (CBF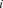). The main circuit breakers must be switched *ON* and the CBB must be switched *OFF* if the transformers are in normal operation. However, if one of these transformers fails, the CBT of the failed transformer must be switched *OFF*, and the CBB is switched *ON* to provide power to the failed transformer feeders.

## Data Collection

Five years data of the electricity distribution company in Baghdad, Iraq, were collected for time between failures (TBFs) of the electric power distribution station. The failure data were recorded manually. To deal with this problem, we reported the number of breakdowns within five years, and also the time between them. This is shown in Table 1. The first column in Table 1 represents failures numbers, and second column mentioned to the number of days require for the station to step-down. For example, the first TBF value was calculated between 12 am on 1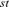 January and 11:50 pm on 14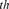 April. Accordingly, the first step-down was occurred after (104.895833).

**Table 1:** Time between failures of the electric power distribution station for five years.

Failure No.	TBFs(day)	Failure No.	TBFs(day)
1	104.895833	2	22.8854167
3	36.729167	4	0.9791667
5	54.8854167	6	83.6875
7	50.895833	8	6.83333
9	97.83333	10	42.8854167
11	149.9791667	12	6.9791667
13	13.9375	14	2.9375
15	70.9791667	16	109.83333
17	36.83333	18	47.78125
19	2.9375	20	118.8020833
21	9.83333	22	529
23	30.895833	24	15.78125
25	71.8854167	26	38.7604167
27	57.8854167	–	–

Each component of the station can be failed in random manner. The component average time between failures was modeled to be random variable following certain distribution [2]. EasyFit is the distribution fitting software that can be used to fit the appropriate statistical distribution for the TBFs. In the next section we will focus on the goodness of fit to find the best fitting statistical distribution for each component of TBFs.

## Goodness of Fit

**Figure 1:**
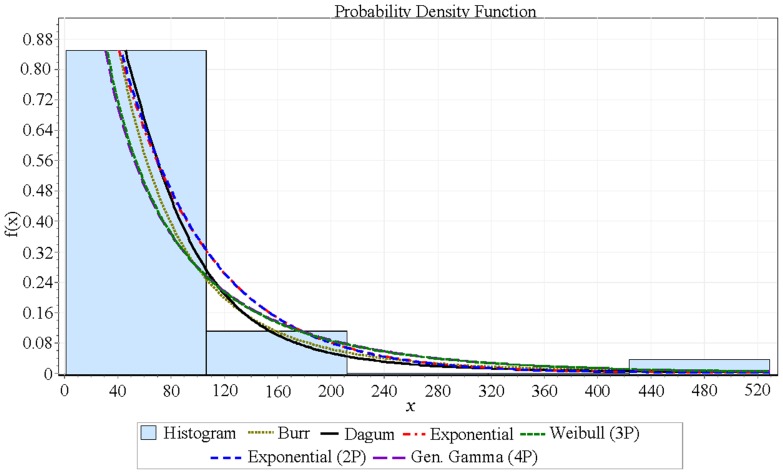
The fitting result for best six distributions of TBFs histogram.

**Figure 2:**
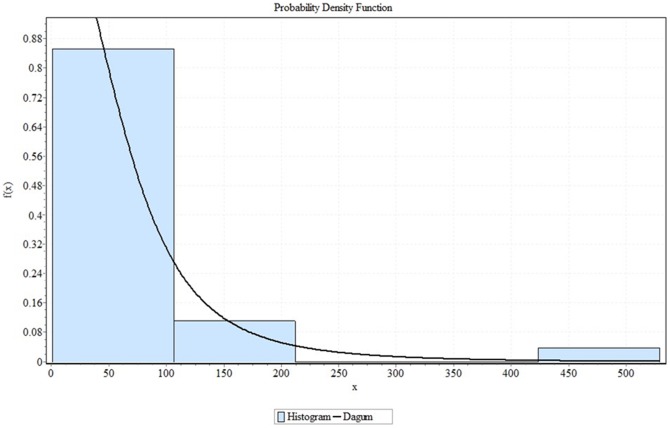
The Dagum distribution fitting result with TBFs data histogram.

**Figure 3:**
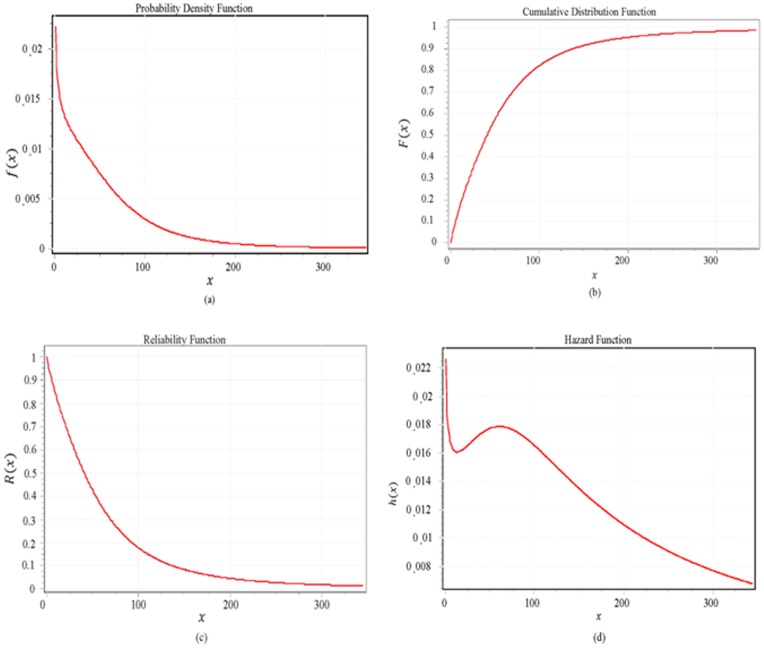
The failure functions of TBFs data of a Dagum random variable with 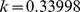
 **,** 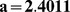 **and** 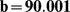 **, (a) Pdf, (b) CDF, (c) Reliability function and (d) Hazard function.**

In this paper a goodness of fit for the TBFs statistical distribution was tested. Such test can be done using many tools. EasyFit software was used to perform this task. It includes the using of the *Kolmogorov-Smirnov*, *Anderson-Darling* and *Chi-square* test. The idea behind the goodness of fit tests is to have the “distance,” critical values, measured between the data and the distribution being tested. Then that critical value is compared to some threshold value. The goodness of fit reports includes the test statistics and the critical values calculated for various significance levels (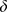 = 0.2, 0.1, 0.05, 0.02, 0.01). Furthermore, the goodness of fit test statistics indicates the distance between the data and the provided distributions [27]. The P-value can be helpful specifically when the null hypothesis is rejected at all selected significance levels, where the P-value is criteria uniformity between the results actually obtained in the experiment and the random chance explanation for those results [28]–[30]. It is required to know at which level it could be accepted [31]. EasyFit deals with data using histogram based on TBFs samples. The number of vertical bars was based on the total number of observations (27 values). The equation 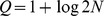, was used to find the number of bins (histogram), where N is the total number of TBFs and Q is the resulting number of classes [32]. The height of each histogram bar indicates how many data points fall into that class. To obtain the best fitting model, we chose various distributions. Our analysis reveals that the distribution with the lowest statistical value is the best-fitting model.Similar conclusion is also drawn by Fakhraei [33], This support the validity of our analysis. Based on this fact, each distribution is ranked (1 =  the best model, 2 =  the next best model and so on). The data was analyzed and tested under several nonnegative distributions using the EasyFit software. Dagum distribution is the optimal analysis of the TBFs, with scale parameter 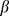 = 90.001 and shape parameters 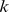 = 0.33998 and 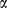 = 2.4011. Table 2 shows the summary of the goodness of fit of TBFs for the (39) nonnegative distributions. Table 3 shows the goodness of fit details of TBFs for Dagum distribution. Figure 1 shows only the six much closer distributions from all 39 nonnegative distributions. Figure 2 shows the fitting result of TBFs histogram with the Dagum distribution while Figure 3 (a, b, c and d) shows the failure functions (Pdf, CDF, Reliability function and Hazard function) respectively, of Dagum distribution (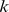 = 0.33998, 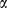 = 2.4011, 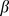 = 90.001).

**Table 2:** The summary of goodness of fit sorted by rank resulting from the *Kolmogorov-Smirnov* test.

	*Kolmogorov Smirnov*		*Anderson Darling*		*Chi-Squared*	
Distribution	Statistic	Rank	Statistic	Rank	Statistic	Rank
Dagum	0.09309	1	0.19582	1	0.49359	10
Exponential	0.09714	2	0.55542	11	1.2128	15
Exponential (2P)	0.09871	3	1.8547	22	0.78094	11
Weibull (3P)	0.11924	4	1.0625	15	0.24672	8
Burr	0.11966	5	0.3036	4	0.11459	4
Gen. Gamma (4P)	0.12057	6	1.1189	16	0.24378	7
Pearson 6	0.12139	7	0.30249	3	0.11222	3
Pareto 2	0.12214	8	0.29996	2	0.16934	5
Frechet (3P)	0.12715	9	0.3937	5	0.82819	12
Gamma (3P)	0.12922	10	4.2307	28	N/A^♠^ 	
Log-Logistic (3P)	0.13296	11	0.51477	10	1.2346	17
Weibull	0.14339	12	0.41791	6	0.42538	9
Burr (4P)	0.1438	13	4.313	29	N/A^♠^ 	
Inv. Gaussian (3P)	0.14394	14	0.44184	7	0.83618	14
Lognormal (3P)	0.1458	15	0.44902	8	1.2296	16
Fatigue Life (3P)	0.14646	16	0.47478	9	0.82997	13
Gen. Gamma	0.15678	17	0.76019	13	1.2862	19
Inv. Gaussian	0.16775	18	2.0065	24	1.2995	20
Lognormal	0.16859	19	0.63295	12	1.2509	18
Gamma	0.18948	20	1.5649	19	1.5286	21
Log-Logistic	0.19874	21	0.83928	14	2.1911	22
Pearson 6 (4P)	0.20203	22	4.7384	30	N/A^♠^ 	
Fatigue Life	0.22105	23	1.299	17	3.883	26
Pearson 5 (3P)	0.22482	24	3.6623	27	0.00149	1
Levy	0.22647	25	1.8258	21	3.4804	24
Levy (2P)	0.22918	26	1.496	18	0.21343	6
Chi-Squared (2P)	0.24256	27	3.1159	25	4.8578	27
Frechet	0.2431	28	1.6912	20	0.01378	2
Rayleigh (2P)	0.24822	29	3.1436	26	3.6554	25
Pearson 5	0.25076	30	2.0032	23	2.3495	23
Rayleigh	0.25405	31	8.3926	32	7.157	28
Pareto	0.31571	32	6.5738	31	8.711	29
Rice	0.45759	33	18.714	33	31.134	30
Chi-Squared	0.5208	34	137.97	35	31.148	31
Dagum (4P)	0.52183	35	19.63	34	62.473	32
Erlang	No fit					
Erlang (3P)	No fit					
Log-Gamma	No fit					
Nakagami	No fit					

^♠^: No answer.

**Table 3:** The details for goodness of fit for a Dagum distribution (3P).

*Kolmogorov Smirnov*					
Sample Size	27				
Statistic	0.09309				
P-Value	0.95633				
Rank	1				
δ	0.2	0.1	0.05	0.02	0.01
Critical Value	0.2003	0.22898	0.25438	0.28438	0.30502
Reject?	No	No	No	No	No
*Anderson Darling*					
Sample Size	27				
Statistic	0.19582				
Rank	1				
δ	0.2	0.1	0.05	0.02	0.01
Critical Value	1.3749	1.9286	2.5018	3.2892	3.9074
Reject?	No	No	No	No	No
*Chi-Squared*					
Deg. of freedom	3				
Statistic	0.49359				
P-Value	0.9203				
Rank	10				
δ	0.2	0.1	0.05	0.02	0.01
Critical Value	4.6416	6.2514	7.8147	9.8374	11.345
Reject?	No	No	No	No	No

## Maximum Likelihood Estimation

The best result of goodness of fitting to the TBFs under many distributions using EasyFit software is the Dagum distribution. Alwan et al. [34] provides a more detailed treatment of the fitting method. The maximum likelihood method is used to estimate the parameters 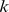, 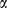, 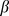 of the Dagum distribution. The likelihood function, 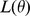, from a generic distribution with density and reliability functions 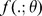 and 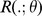, respectively, can be written as [9].
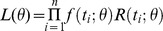



where 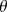 is the parameter(s) of the distribution and 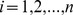. Consider a sample of size n (which is 27 samples in this paper). The log-likelihood function for the estimate of the parameters 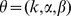 of the Dagum distribution is given by Domma et al. [9]. That is, the log-likelihood function, 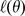, based on data from Eq.1 is [9].



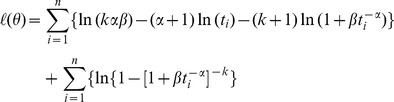
(5)The MLEs 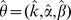 are obtained from the numerical maximization of Eq.5, since the solution of the maximum likelihood equations is not in closed form [9]. Using Eq.5 the values of the estimated 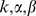 parameters for each component of the station relying on the maximum likelihood method are presented in Table 4.

**Table 4:** Estimated scale and shape parameters of Dagum distribution for each component.

components	*K*	*α*	*β*
T 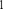	0.1319	4.0147	111.0092
T 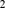	0.1283	5.5402	163.794
CBT 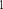	20.8619	0.6425	0.110026
CBT 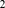	0.0764	12.2022	120.841
CBF 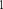	12.4569	1.18365	3.75407
CBF 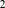	2.02372	1.24288	13.0104
CBF 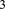	0.69703	1.45807	32.3148
CBF 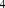	0.929475	1.23822	21.3319
CBF 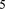	1.83028	1.20514	14.535
CBF 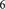	178.257	1.3475	0.403921
CBF 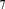	9.52767	0.3868	0.01772
CBF 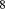	1.37434	1.53844	37.4659
CBF 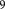	1.92766	1.45856	12.6204
CBF 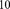	1.50505	0.18518	14.0558

## Reliability Assessment

**Figure 4:**
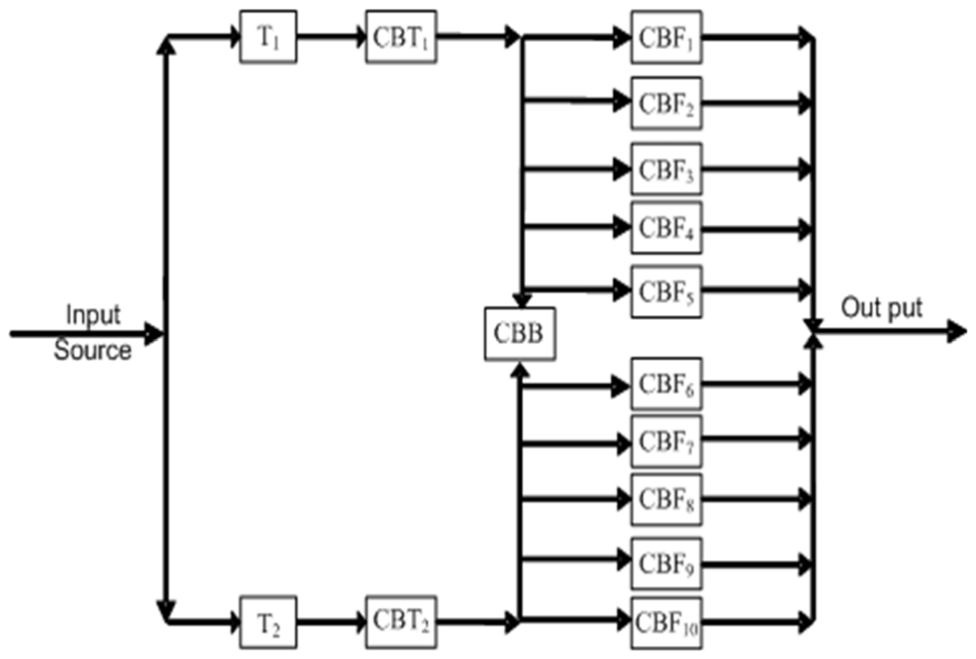
The reliability block diagram of the 33/11 KV electric power distribution station.

Figure 4 shows the reliability block diagram for the electric power distribution station. It also represent the visualization of the components working. The reliability function for a Dagum random variable was provided in Eq.3. In the section Problem Statement, the CBB does not function if, and only if, one of the transformers do not operate. Fourteen different components exist, excluding the CBB. Based on Figure 4, the following classifications of the block diagram reliability of the system for the electric power distribution station have been described as

### First Group(FG)

Transformers 1 and 2 as well as the main circuit breaker are connected together in a series. At the same time, the two transformers (Transformers 1 and 2) and their main circuit breaker are connected in a parallel manner (see Figure 4). The reliability function of this group is expressed as
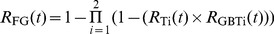
(6)


where 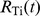 and 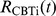 are the reliability of transformer i and of the main circuit breaker of transformer *i*, respectively, during the period *t*.

### Second Group (SG)

The feeders are connected in a parallel manner, indicating that
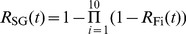
(7)


where 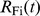 is the reliability of feeder *i* during the period of *t*.

The group of transformers and the group of feeders are connected in a series. The reliability function of the system is
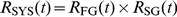
(8)


Based on the values presented in Table 4 and by using Eqs. 3, 6, 7, and 8, we can calculate the system reliability for the times imposed from *t*  = 1 to *t*  = 30, where *t* is expressed in days. The data are presented in Table 5.

**Table 5:** Estimated reliability system values of the electric power distribution station for 30 days.

T(day)	*R_SYS_*	T(day)	*R_SYS_*
1	0.996500665	2	0.989498957
3	0.979827045	4	0.968407728
5	0.95585446	6	0.942558222
7	0.928774489	8	0.914675671
9	0.900381613	10	0.885977738
11	0.871526224	12	0.857073115
13	0.842652974	14	0.828292031
15	0.814010334	16	0.799823272
17	0.785742656	18	0.771777517
19	0.75793469	20	0.744219264
21	0.730634929	22	0.717184252
23	0.703868901	24	0.690689829
25	0.67764743	26	0.664741662
27	0.651972165	28	0.639338348
29	0.626839464	30	0.614474679

## Limitations of the Study, Open Questions, and Future Work

We believed that the limitations are:

- For the sake of brevity, we restrict our investigation to one electric power distribution station.
- We have used the data for five consecutive years.
- We used EasyFit software for our investigation.
- The study focused in details inside the electric power distribution station, without return to the source. Note that if the source feeds the electric power distribution station by low energy (less than 33 kilovolt), this leads to a high temperature in the transformer which will cause a sudden stop of power station.

The present paper deals with the electric power distribution station as independent and separate components. If one take the data of failure rate (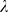) for each components of this station and deal with 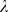 by using the Markov model “Hidden Markov model.” Then one may get better performance of the electric power distribution station. It is know that the Markov model dependent on the current state of the failure, rather returning to the history of the data [35].

The current paper may be extended to scrutinize preventive maintenance modelling and to estimate its effects on the components of the station. This might improve the supply of electrical energy and will reduce the operating cost of the power station.

## Conclusion

The time between failures was analyzed to determine the best-fitting distribution. Using the distribution fitting software EasyFit, we determined that the most valid distribution is the Dagum distribution with a scale parameter 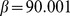, shape parameters 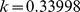 and 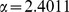. The reliability value for the system on the first day was 0.99. If the station works for 30 days, the reliability value of station was decreased to 0.61. The value of the reliability function was declined by 38.2% in 30 days. This percentage indicates that the electric power distribution station studied in this paper exposed to fail close together in time, even in the same part. This leads to two possibilities; the first is that, the maintenance staff and engineers are not doing their work at best performance, the second is that parts for the electric power distribution station, consisting of 14 components are not good and are exposed to crash shortly after repairs. The first reason can be ignored, because, the field visits by researcher can emphasize the expertise of engineers and maintenance workers in the completion of repairs and maintenance in record time and dynamically good as mentioned in the records. The main problem is that the sum of component is not good. With reference to the case of calculating the Eqs. 6 and 7 for the reliability value for each component, it can be seen that the items CBT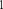, CBF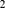, CBF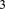, CBF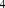, CBF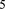, CBF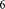, CBF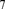 and CBF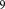 have the largest percentage in the value of reliability. Their reliability values after 30 days become as, 0.42, 0.45, 0.40, 0.37, 0.47, 0.41, 0.40, and 0.38 respectively. These values show that the components of the electric power distribution station continuously deteriorate because of aging. Furthermore, these components CBT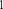, CBF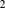, CBF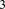, CBF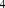, CBF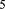, CBF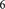, CBF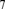 and CBF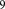 had the highest critical value and must be changed with new items as soon as possible.
